# Enhancing Predicted Efficacy of Tumor Treating Fields Therapy of Glioblastoma Using Targeted Surgical Craniectomy: A Computer Modeling Study

**DOI:** 10.1371/journal.pone.0164051

**Published:** 2016-10-03

**Authors:** Anders Rosendal Korshoej, Guilherme Bicalho Saturnino, Line Kirkegaard Rasmussen, Gorm von Oettingen, Jens Christian Hedemann Sørensen, Axel Thielscher

**Affiliations:** 1 Department of Neurosurgery, Aarhus University Hospital, Aarhus, Denmark; 2 The Danish Research Centre for Magnetic Resonance, Copenhagen University Hospital Hvidovre, Hvidovre, Denmark; 3 Max Planck Institute for Biological Cybernetics, Tübingen, Germany; 4 Biomedical Engineering, DTU Elektro, Technical University of Denmark, Kongens Lyngby, Denmark; Wake Forest University School of Medicine, UNITED STATES

## Abstract

**Objective:**

The present work proposes a new clinical approach to TTFields therapy of glioblastoma. The approach combines targeted surgical skull removal (craniectomy) with TTFields therapy to enhance the induced electrical field in the underlying tumor tissue. Using computer simulations, we explore the potential of the intervention to improve the clinical efficacy of TTFields therapy of brain cancer.

**Methods:**

We used finite element analysis to calculate the electrical field distribution in realistic head models based on MRI data from two patients: One with left cortical/subcortical glioblastoma and one with deeply seated right thalamic anaplastic astrocytoma. Field strength was assessed in the tumor regions before and after virtual removal of bone areas of varying shape and size (10 to 100 mm) immediately above the tumor. Field strength was evaluated before and after tumor resection to assess realistic clinical scenarios.

**Results:**

For the superficial tumor, removal of a standard craniotomy bone flap increased the electrical field strength by 60–70% in the tumor. The percentage of tissue in expected growth arrest or regression was increased from negligible values to 30–50%. The observed effects were highly focal and targeted at the regions of pathology underlying the craniectomy. No significant changes were observed in surrounding healthy tissues. Median field strengths in tumor tissue increased with increasing craniectomy diameter up to 50–70 mm. Multiple smaller burr holes were more efficient than single craniectomies of equivalent area. Craniectomy caused no significant field enhancement in the deeply seated tumor, but rather a focal enhancement in the brain tissue underlying the skull defect.

**Conclusions:**

Our results provide theoretical evidence that small and clinically feasible craniectomies may provide significant enhancement of TTFields intensity in cerebral hemispheric tumors without severely compromising brain protection or causing unacceptable heating in healthy tissues. A clinical trial is being planned to validate safety and efficacy.

## Introduction

Glioblastoma Multiforme (GBM) is one of the most severe and debilitating types of brain cancer. The current standard of GBM therapy involves surgical tumor resection, radiotherapy, and chemotherapy [1–7]. Within recent years, however, tumor treating fields (TTFields) has become increasingly common as a supplementary treatment modality in several neuro-oncology centers around the world. Results have been promising [8–17] with efficacy being comparable to best choice physicians chemotherapy for recurrent GBM [9,10,13,18] and overall survival time increased by approximately 5–7 months for newly diagnosed GBM [13,19]. Despite recent improvements, no treatment to date has been able to substantially increase the chance of long-term survival for GBM patients and the disease remains a deadly condition.

In this study, we present a new clinical implementation of TTFields therapy, which has the theoretical potential to significantly increase the clinical efficacy of TTFields therapy of intracranial cancers. Standard TTFields therapy utilizes intermediate frequency (100–300 kHz) alternating currents to disrupt cancer cell division and arrest tumor progression [20,21]. *In vitro* studies have established that the effect of TTFields on tumor growth rate is positively correlated to the electrical field strength induced by the treatment [20,22]. Although an equivalent correlation between electrical field strength and clinical tumor control has not been firmly established it is likely that this dose-response relationship extends to clinical settings. From a clinical point of view, it is therefore desirable to apply TTFields therapy in a way which focally increases the field strength in the tumor region while sparing healthy regions of the brain. Here we propose an intervention which achieves this objective for tumors in the cortical and subcortical regions of the cerebral hemispheres. The approach combines TTFields therapy with removal of selected parts of the skull (craniectomy) immediately over the tumor region in order to create low resistance pathways for the current flow into the underlying tumor tissue. We investigated the estimated efficacy of various sizes and configurations of craniectomy with the objective to identify approaches which are effective, safe and feasible from a clinical perspective. In addition, we investigated the efficacy of the intervention for a deeply seated tumor and addressed the potential limitations in the range of anatomical applicability. We used finite element methods to calculate the electrical field distribution induced by TTFields, both in a standard configuration (no craniectomy) and following a variety of virtual surgical craniectomies located immediately above the tumor region. We quantified the expected field enhancement induced by this approach to obtain a surrogate measure of the expected enhancement in treatment efficacy [23]. To illustrate the most likely clinical scenarios we have investigated the expected impact both before and after the resection of the superficial tumor. Based on the *in vitro* dose-response relationship between field strength and decline in tumor growth rate [20,22], the proposed implementation has the theoretical potential to considerably increase TTFields efficacy for glioblastoma patients without compromising brain protection and patient safety to an unacceptable extent. The potential benefit expectedly applies to a wide range of tumor locations in the cerebral hemispheres, although only limited effect can be expected for very deep tumors located in the thalamic regions and basal ganglia. Our results lay the grounds for future pre-clinical and clinical studies to validate the concept.

## Materials and Methods

The study protocol was submitted for approval by the Central Denmark Region Committee for Health research Ethics. The study was accepted and full review was not required, as the study was not considered to be a clinical research project. Use of clinical MRI scans for electrical field modeling studies was approved. The study was performed in accordance with the principles expressed in the Declaration of Helsinki [24] and written consent to use MRI data for the study was obtained from all included subjects.

### Study subjects

**Fig 1:**
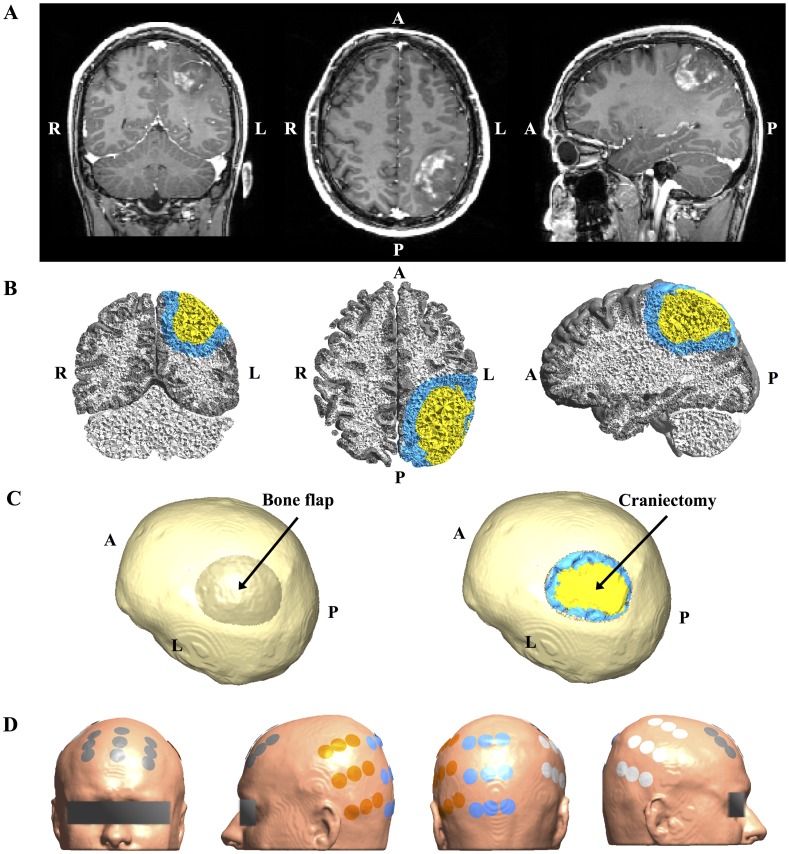
MRI data from study Subject 1 and corresponding 3D head model. **A.** Coronal (left), axial (middle) and sagittal (right) views of original Gadolinium enhanced T1 MRI patient data showing left parietal glioblastoma (radiological orientation). **B.** Volume reconstruction of gray matter (gray), white matter (white), tumor tissue (yellow), and a peritumoral region (blue). **C.** Surface reconstruction of patient skull rotated to present the craniectomy boneflap outlined as a darkened area above the tumor (left). The rightmost image shows the same view, but with the bone flap removed to display the underlying tumor and peritumoral region. **D.** Surface reconstruction of the head model showing the optimized electrode layout used for simulation (NovoTAL ^™^). Electrodes are paired orange with white and gray with blue.

**Fig 2:**
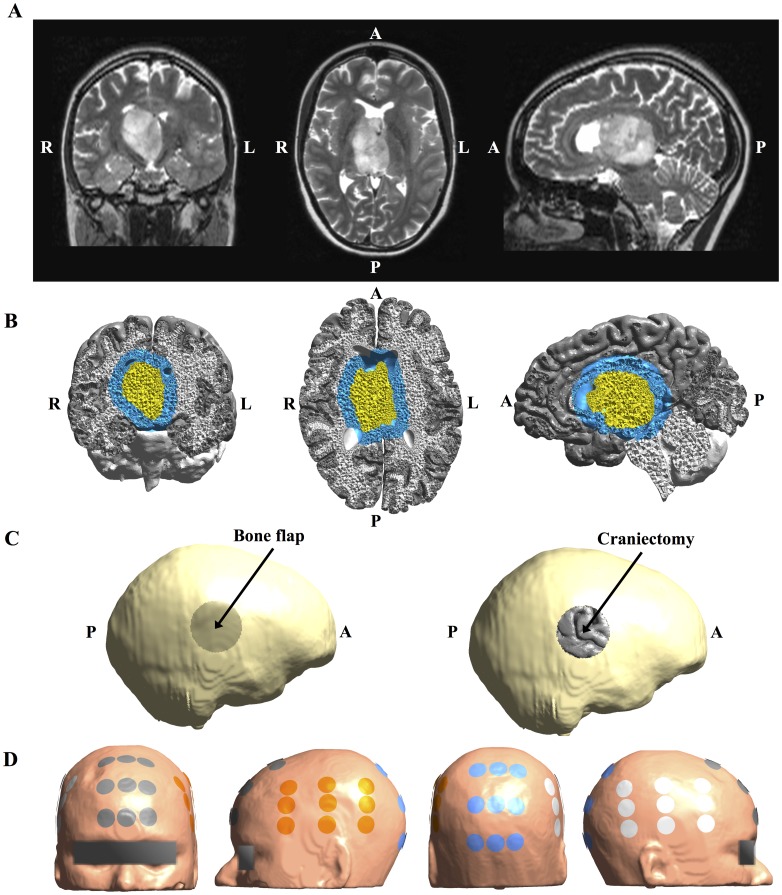
MRI data from study Subject 2 and corresponding 3D head model. **A.** Coronal (left), axial (middle) and sagittal (right) views of original T2 MRI patient data showing a deeply seated WHO III astrocytoma (radiological orientation). The tumor was non-enhancing on T1 with Gadolinium enhancement. **B.** Volume reconstruction of gray matter (gray), white matter (white), tumor tissue (yellow), and a peritumoral region of interest (blue). **C.** Surface reconstruction of patient skull rotated to present the circular 50 mm craniectomy boneflap outlined as a darkened area above the tumor on the right side. The rightmost image shows the same view, but with the bone flap removed to display the underlying cortical surface. **D.** Surface reconstruction of the head model showing the optimized electrode layout used for simulation (NovoTAL^™^). Electrodes are paired orange with white and gray with blue.

Experiments were based on MRI data obtained from two patients: 1) A 36-year-old patient (Subject 1) with a 50x35x51 mm hemispheric left fronto-parietal GBM (Fig 1A) centered at approximately 20 mm depth relative to the cerebral cortex, and 2) a 22-year-old patient (Subject 2) with a deeply seated a 32x51x41 mm WHO grade III astrocytoma located in the right thalamus and surrounding basal ganglia and white matter tracts (Fig 2A). The choice of patients was based on the objective to investigate the potential benefit, applicability and limitations of the proposed skull remodeling procedure for both superficial hemispheric and deeply seated tumors and further in order to illustrate the expected mechanism of action of the intervention. Subject 1 is representative of most GBM tumors in the sense that it is located in the hemispheric region and has a representative size and radiological appearance. Studies of GBM volume distributions in the brain have shown that approximately 97% of GBM tumors are located in the cerebral hemispheres with significant proportions of the tumor volume located in the superficial parts of the hemispheres [25]. Deep locations such as the basal ganglia or thalamic regions are very uncommon. This observation is relevant for the present applications as our results demonstrate that the intervention will arguably benefit most GBM tumors with significant volume proportions located in the most superficial 4–5 centimeters of the cerebral hemispheres (see Results and Discussion). Contrary to the representative Subject 1, Subject 2 represents a rare case of deeply located GBM. For these reasons detailed investigations of the impact and utility of craniectomy were based on data from Subject 1, while data from Subject 2 was mainly included to illustrate limitations in applicability of the skull remodeling procedure with regards to anatomical range of tumor location. With regards to Subject 2, it should be noted that anaplastic astrocytoma is not an approved indication for TTFields therapy. However, the disease bares significant similarities to GBM, such as similar cellular origin, and it is considered a highly malignant tumor with a poor prognosis. The chemo- and radiotherapy regimen for anaplastic astrocytoma is most often the same as for GBM and in many cases anaplastic astrocytoma will dedifferentiate to GBM. Furthermore, when viewed from a modeling perspective, the tumor may be considered as equivalent to GBM, since the conductivity distributions of the two tumor types are known to be highly comparable based on MRI based conductivity estimation and *in vitro* measurements [26–30] and these findings were confirmed for the presented cases (see below).

### Finite element head models

A finite element head model was generated for each patient based on T1 and T2 MRI data using a custom version of the SimNIBS pipeline (www.simnibs.org) [31,32]. For statistical purposes, we defined a peritumoral volume of interest, including all finite elements with centers closer than 1 cm to the tumor border, as exemplified in Fig 1B. For Subject 1 a realistic craniotomy was outlined manually in the skull above the superficial part of the tumor (Fig 1C) and modeled by assigning isotropic skin conductivity values (0.465 S/m, see below) to finite elements belonging to the outlined bone flap. This was equivalent to virtually replacing the bone flap with skin tissue. Using a similar approach, circular craniectomies with diameters ranging from 10 to 100 mm (5 mm increments) were also introduced immediately above the tumor to assess the importance of craniectomy size. Finally, a model with four 15 mm burr holes distributed across the tumor projection on the skull was created to investigate differences in performance between two types of craniectomy with the same area but different configuration. In order to investigate realistic treatment scenarios, gross total tumor resection was also modeled for Subject 1 by assigning CSF conductivity values to all tumor elements, thereby creating a virtual resection cavity filled with CSF. This situation, in which the resection cavity is filled with CSF, is representative of the majority of TTFields exposure time for patients who have undergone surgical resection. This is due to the fact that TTFields is always initiated at least four weeks following surgery at which time blood and hemostatic compounds, which have potentially been introduced in the resection cavity at the time of surgery, will have dissolved and become replaced by CSF.

For Subject 2, a circular craniectomy of 5 cm diameter was placed beneath the closest TTFields generating electrode patch, which was located on the right side of the head (Fig 2C). This patient was not eligible for surgery and thus only the clinically relevant situation, in which the tumor was present *in situ* and no resection was performed, was investigated.

### Electrical field calculation and electrode design

Electric field calculations were performed using SimNIBS [31–34]. Finite element methods were applied to obtain a numerical solution to Laplace’s Equation of the electrostatic potential. The model was designed to represent a realistic treatment setting, i.e. electrode design was equivalent to the Optune^™^ technology (private communication with Novocure^™^ Ltd.) [31,35–39]. Calculations were based on a peak-to-peak current amplitude of 1.8 A in each pair of electrode arrays and the estimated electrical field distributions thereby represented the distribution of the *maximum* field intensity which occurs at one point during a duty-cycle period (5 μs) when the alternating current reaches peak amplitude. It is thereby a repeating snapshot of the field distribution, which occurs repeatedly every 5 μs (200 kHz) throughout a patient’s continuous exposure to TTFields (> 18 hours per day). The calculated field will scale linearly with the current amplitude throughout the remainder of the duty cycle. Since the distribution is determined by the anatomy and electrical properties of the patient’s head, in combination with the geometry, location and settings of the active electrodes, it can be assumed that the estimated field distribution will remain representative of the treatment throughout the period of exposure as long as the radiological images used for simulation are representative. Clearly, dynamic changes may occur in the region of pathology, such as progression or regression of the tumor, and such cases should be regarded as separate scenarios for which separate field calculations should be performed to obtain the highest possible accuracy.

Layouts of electrodes on the scalp (Figs 1D and 2D) were designed for the both patients using the NovoTAL^™^ software [40], which is used for clinical treatment planning (courtesy of NovoCure^™^). The setup consisted of two sequentially activated separate current sources connected to a left-right (L/R) and anterior-posterior (A/P) electrode array pair, respectively. Anisotropic conductivity estimates of intracranial tissues were obtained from diffusion tensors measurements using a direct mapping scheme [31,41–43]. The slope of the linear fit was optimized so that the mean squared error between the estimated conductivities in gray and white matter of the unaffected right hemispheres and the respective literature values of 0.275 S/m and 0.126 S/m were minimized. For Subject 1 the resulting distributions had median (*Q*_2_) and interquartile range (IQR) values of $Q_{2}^{gm}=0.186\;S/m\;(IQR_{gm}=0.059\;S/m)$ and $Q_{2}^{wm}=0.159\;S/m\;(IQR_{wm}=0.031\;S/m)$, respectively, and were thus close to the corresponding literature values. The median conductivity in the tumor was $Q_{2}^{tumor}=0.244\;S/m\;(IQR_{tumor}=0.077\;S/m)$, which also corresponds well to *in vivo* measurements of 0.10−0.43 *S*/*m* obtained at comparable frequencies [26]. For Subject 2 the corresponding values were $Q_{2}^{gm}=0.195\;S/m\;(IQR_{gm}=0.069\;S/m)$, $Q_{2}^{wm}=0.178\;S/m\;(IQR_{wm}=0.033\;S/m)$, and $Q_{2}^{tumor}=0.220\;S/m\;(IQR_{tumor}=0.066\;S/m)$ and thus also within the expected range. Conductivities of CSF, skin, and bone were considered isotropic and assigned corresponding *in vivo* estimates of 1.654 S/m, 0.465 S/m and 0.010 S/m [26,44–47].

### Estimation of clinical efficacy

Expected clinical efficacy was assessed by calculating the therapeutic enhancement ratio (TER) for each element in the tumor and peritumoral region. TER measures the change in tumor growth rate caused by TTFields (*GR*_*TTF*_) relative to control tissue (*GR*_*c*_) and is defined as TER = (*GR*_*c*_−*GR*_*TTF*_)/*GR*_*c*_ [20,21]. TER > 0 represents reduced growth rate of GBM tissue exposed to TTFields and TER > 1 implies tumor regression. TER was calculated from the electrical field distribution using the relationship inferred from *in vitro* data obtained from human glioma (U-118, U-87) and rat glioma (F-98, C-6, and RG2) cell lines exposed to TTFields at 200 kHz presented in Kirson et al. [21]. An equivalent relationship has not been investigated for glioma cultures based directly on patient specimens. The data was fitted using a third order polynomial, *p*(*x*) = *p*_0_ + *p*_1_*x* + *p*_2_*x*^2^ + *p*_3_*x*^3^ and the resulting linear parameters were *p*_0_ = 4.06·10^−7^, *p*_1_ = −1.72·10^−4^, *P*_2_ = 2.96·10^−2^, and *p*_3_ = −1.54. In order to ensure conservative assessment of treatment efficacy, only values within the range covered by the measurements of Kirson et al. [21] (110 V/m– 240 V/m) were interpolated using the above polynomial regression. Field values below 110 *V*/*m* were assigned a TER value of zero, while values above > 240 *V*/*m* were assigned the value *TER* = 1.245 corresponding to the TER for 240 *V*/*m*. Percentages of tumor and peritumoral volumes in growth arrest or regression were calculated as P_225_ = Prob(|**E**| ≥ 225 V/m), equivalent to Prob(TER ≥ 1). The percentage of pathological tissue in which the growth rate was expectedly reduced by TTF was calculated as P_100_ = Prob(|**E**| ≥ 100 V/m), equivalent to Prob(TER > 0) [40].

## Results

### Effect of craniectomy without tumor resection

**Fig 3:**
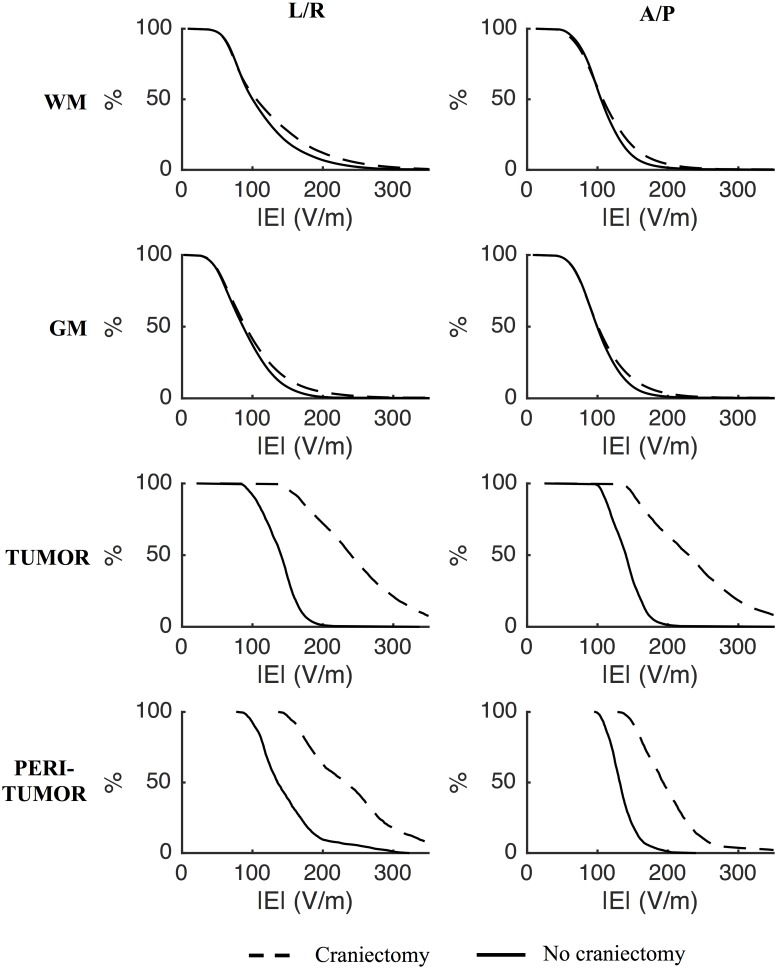
Effect of craniectomy without tumor resection. Percentage of tissue exposed to field strengths above the corresponding value on the abscissa (craniectomy—stippled line; no craniectomy—solid line). Rows represent different tissues and columns the L/R and A/P electrode pairs, as indicated. Craniectomy significantly increased the electrical field strengths in tumor tissue and the peritumoral region compared to no craniectomy. The distributions of field strengths in healthy tissues were largely unaffected.

**Fig 4:**
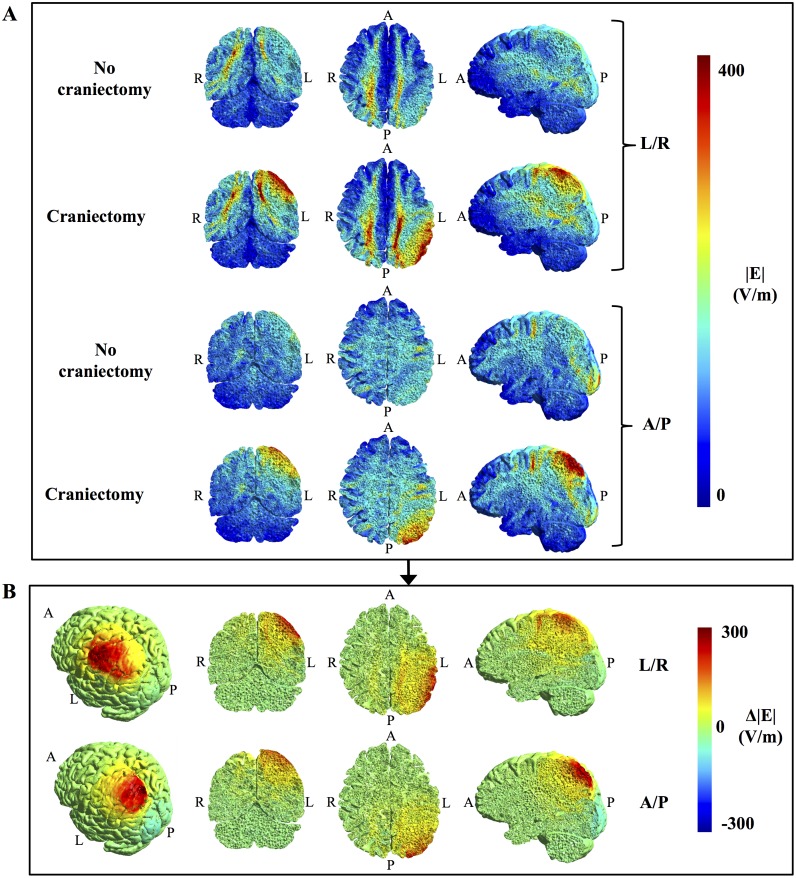
Topographical effect of craniectomy without tumor resection. **A.** Field strength distributions with and without craniectomy (coronal, axial, and sagittal sections from left to right, colorbar 0–300 V/m). **B**. Paired difference between craniectomy and no craniectomy scenarios, i.e. Δ|**E**| = |**E**|_craniectomy_—|**E**|_no craniectomy_, for both electrode pairs. Leftmost panels show a rotated surface view of the region of pathology. Craniectomy produced a marked and focal increase in electrical field strength in the regions of pathology underlying the craniectomy, while healthy tissues were largely unaffected.

Initial analysis was performed on data from Subject 1 and based on a simple scenario in which a realistic craniotomy bone flap was virtually removed and replaced by skin (will be referred to as *standard craniectomy*). This type of craniectomy may be obtained by simply removing the bone flap created in connection with primary surgical tumor removal. In the present case, the craniectomy was approximately oval with principal diameters of 50 mm and 67 mm, respectively, and a total surface area of 2902 mm^2^. The cumulative distributions of electrical field strengths obtained before and after standard craniectomy are presented in Fig 3 for each tissue. The topographical maps of the same estimates are presented in Fig 4A along with the topographical distribution of the paired difference between them (Fig 4B). The results generally illustrate that craniectomy produced a marked and focal enhancement of treatment efficacy in the regions of pathology underlying the craniectomy without inducing high electrical field strengths in the surrounding healthy tissues.

In the tumor volume, craniectomy caused a paired median increase in field strength of 95 V/m (corresponding to 68%) for the L/A electrode array pair and 85 V/m (61%) for the A/P pair, relative to the standard situation with no craniectomy. The absolute median field strengths following craniectomy were 240 (IQR = 96) V/m for the L/R pair and 230 (IQR = 104) V/m for the A/P pair. Craniectomy increased the field strength in nearly >95% of the tumor volume, indicating that treatment efficacy was improved in nearly the entire region of pathology. The percentage of tumor tissue in expected growth arrest or regression was increased from negligible values (P_225, tumor_ < 0.5%) before craniectomy to significant proportions after craniectomy (P_225, tumor_ = 59% for the L/R pair and P_225, tumor_ = 52% for the A/P pair). Following craniectomy, more than 99.5 percent were exposed to field strengths higher than the minimum threshold value of 100 V/m and mean TER values were increased from ~ 0.37 (L/R and A/P) to 1.13 for the L/R pair and 1.04 for the A/P pair. The latter observation suggests that craniectomy may potentially convert a situation with considerable tumor progression despite TTFields therapy into a situation with average *growth regression* at a rate of 4–13% of the normal glioblastoma growth rate. This marked increase in TTF efficacy highlights the potential clinical impact of the procedure.

Compared to the tumor volume, similar beneficial tendencies of craniectomy were observed in the peritumoral region although the absolute effect was slightly less pronounced. The median increase in field strength induced by the L/R pair was 85 V/m (63%), while the A/P pair caused a 58 V/m (44%) increase. Equivalently P_225_ was increased from 7% to 53% for the L/R pair and from 0 to 23.5% for the A/P pair. Mean TER values increased from 0.39 to 1.03 for the L/R pair and from 0.31 to 0.81 for the A/P pair.

In healthy gray and white matter the distributions of field strengths were largely unaffected. In addition, the median specific absorption rates (SAR = σ|**E**|^2^/ρ, σ: tissue conductivity, ρ: tissue density) of skin tissue in the regions underlying the electrodes and overlying the craniectomy were also unaffected by the intervention (SAR_pre_ = 4–6 W/kg and SAR_post_ = 5–7 W/kg). This indicates that craniectomy would not cause unacceptable heating of healthy tissues in the presented case. Peak SAR values (99^th^ percentiles) in the same regions were in the range 38–53 W/kg before craniectomy and 54–83 W/kg following craniectomy and these results are all in the range of previously reported *median* values obtained using FEM modeling with no craniectomy [35]. Although this also supports acceptable safety of the procedure, the fact that craniectomy did increase peak SAR values does indicate a higher risk of localized adverse skin effects following craniectomy and this circumstance should be observed in clinical implementations.

**Fig 5:**
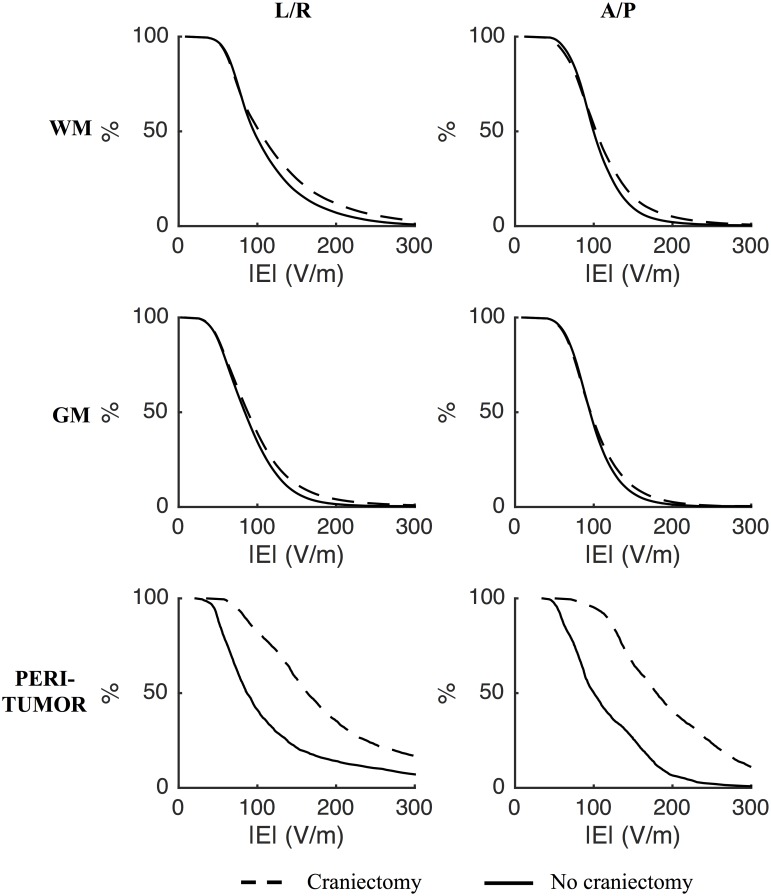
Effect of craniectomy on current density. **A.** Current density distribution (color indication 0–180 A/m^2^) on the brain surface with and without craniectomy (no resection). The skin surface (with placed electrodes) is shown for orientation. Craniectomy significantly increases the current density in the region of pathology underlying the craniectomy (black ellipse, Fig 1C). This in turn leads to increased field strength in the affected region. **B**. Topographical distribution of the current density on the skin surface with and without craniectomy (range 0–250 A/m^2^). Craniectomy causes the current to be shunted through the skull defect thereby lowering the current density in the skin region between the active electrodes. The figure also shows how individual electrodes in the arrays contribute differently in the two situations.

It is noticeable that craniectomy produced a relatively focal enhancement of the electrical field strength in the underlying region, as previously described for tDCS [23]. This resulted from the fact that craniectomy created a corridor for high current flow into the underlying intracranial tissue, as illustrated in Fig 5A. In addition, curre<nt density in the skin between electrodes was reduced (Fig 5B) due to shunting through the hole in the skull. Craniectomy thereby redistributed the current flow to pass through the region of pathology and thereby also caused clear-cut changes in the topographical distribution of electrical field strength in this region (Fig 4). The corresponding coefficients of determination between the pre- and post-craniectomy scenarios were r^2^_tumor, L/R_ = 0.53, r^2^_tumor, A/P_ = 0.64, r^2^_peritumor, L/R_ = 0.73, and r^2^_peritumor, A/P_ = 0.37. In light of the mechanism of action indicated above, craniectomy should be considered a method to focally intensify TTFields in the underlying region. Particularly, it should be noted that the ability of a craniectomy to selectively enhance the field intensity in the region of pathology in the presented case was attributed mainly to the fact that the skull defect and electrodes were placed immediately over the tumor region.

### Effect of craniectomy after tumor resection

**Fig 6:**
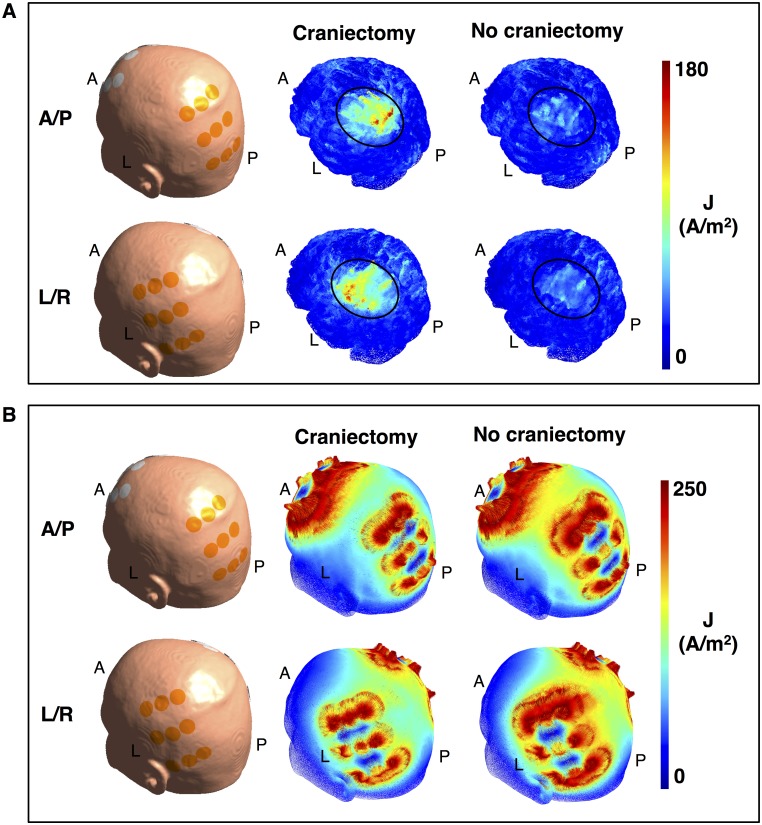
Effect of craniectomy after tumor resection. Percentage of tissue exposed to field strengths above the corresponding value on the abscissa (craniectomy—stippled line; no craniectomy—solid line). Rows represent different tissues and columns the L/R and A/P electrode pairs, as indicated. Craniectomy significantly increases the field strength in the peritumoral region compared to the situation with no craniectomy. The field strengths in healthy tissues were largely unaffected.

**Fig 7:**
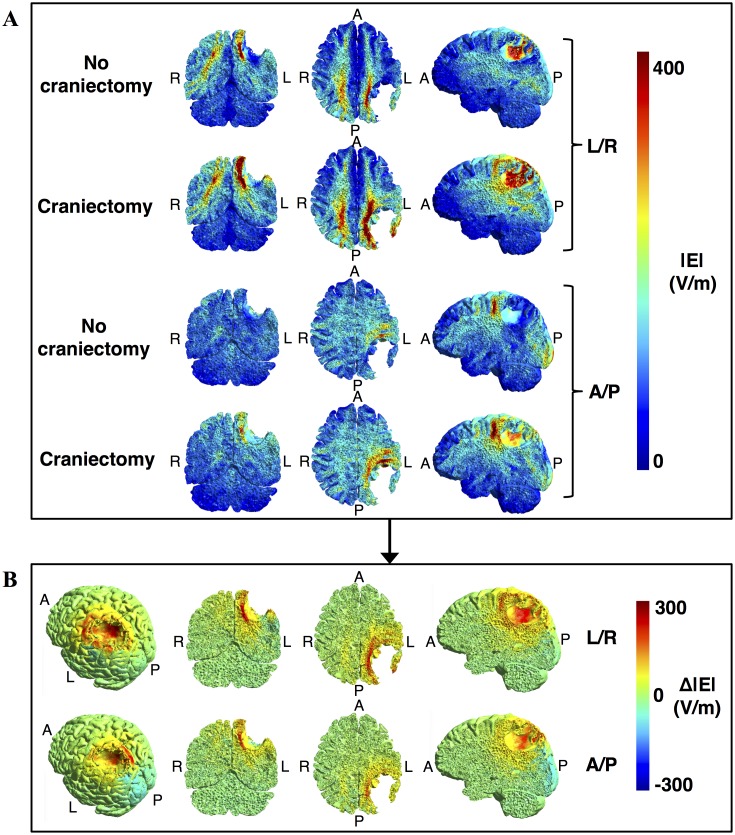
Topographical effect of craniectomy after tumor resection. **A.** Topographical maps of field strength distributions (coronal, axial, and sagittal from left to right, colorbar 0–300 V/m) with and without craniectomy and for both electrode pairs as indicated. **B**. Paired difference between craniectomy and no craniectomy scenarios, i.e. Δ|**E**| = |**E**|_craniectomy_—|**E**|_no craniectomy_, for both electrode pairs. Leftmost panels show a rotated surface view of the resection cavity and the surrounding region of pathology. Craniectomy produced a marked and focal increase in electrical field strength in the peritumoral region underlying the craniectomy, while leaving the healthy tissues largely unaffected.

Following resection of the tumor, standard craniectomy produced a qualitatively comparable enhancement of treatment efficacy, although the absolute effect (Fig 6) and topographical field distribution were significantly different from the preresection results (Fig 7).

The median paired change in peritumoral field strength caused by craniectomy was 83 V/m (96%) for the L/R electrode array pair and 58 V/m (58%) for the A/P pair. Following craniectomy the median field strength was 233 V/m (IQR = 98 V/m) for the L/R pair and 193 V/m (IQR = 56 V/m) for the A/P pair. P_100_ was approximately doubled and >85% for both pairs following craniectomy, while P_225_ increased from 12% to 28% for the L/R pair and from 4% to 32.5% for the A/P pair. Mean TER values were increased from 0.23 to 0.58 for the L/R pair and from 0.21 to 0.68 for the A/P pair. Considering the latter results, craniectomy would thereby reduce cancer proliferation rate in the peritumoral tissue from approximately 80% to 30–40% of the normal glioblastoma growth rate. The topographical field distribution was generally less affected by craniectomy in the post-resection scenario compared to the pre-resection scenario, although some changes did occur (Fig 7, r^2^_peritumor, L/R_ = 0.92 and r^2^_peritumor, A/P_ = 0.80).

Equivalent to the preresection scenario, craniectomy induced a focal enhancement of the field strength in most of the peritumoral region, while not affecting surrounding healthy tissues significantly. SAR values in the skin regions underlying the electrodes and overlying the craniectomy were unaffected by the procedure.

### Importance of craniectomy size

**Fig 8:**
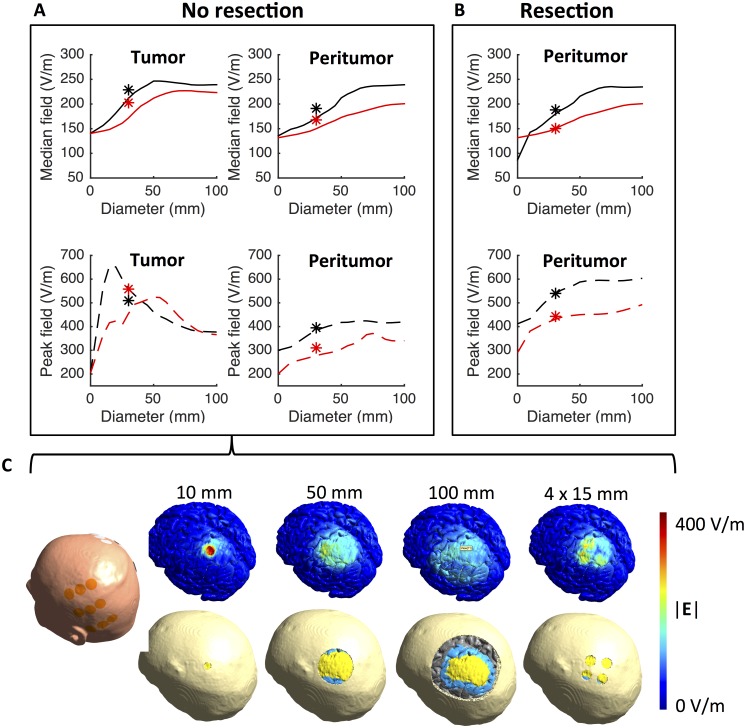
Effect of craniectomy size and configuration on field distribution. **A.** Median and 99^th^ percentile field strengths (*ordinate*) in the tumor and peritumoral tissues at different craniectomy diameters (*abscissa*). Results are shown for both the L/R (red line) and A/P (black line) array pairs. Asterisk symbols represent equivalent results obtained using a model with four 15 mm burr holes located above the tumor region. The results are displayed at 30 mm craniectomy diameter as these configurations had the same total area. **B.** Equivalent results as displayed in A but after resection of the tumor. **C.** Surface view of selected craniectomies and the corresponding field distributions obtained before tumor resection. Color bar represent the range of field strengths displayed.

In order to assess the impact of craniectomy size we calculated the electrical field distribution using circular craniectomies ranging from 10 to 100 mm (5 mm increments). All craniectomies were centered at the same position immediately above the tumor region (Fig 8C). The range of craniectomies spanned from small burr holes, which are clinically feasible to implement without compromising brain protection, to very large craniectomies, which are highly dangerous to implement without external brain protection. Although only some of the investigated craniectomies are feasible from a clinical point of view, the wide range of diameters was chosen to assess whether an optimum size may be determined for the individual patient. In order to assess expected efficacy and safety at each diameter, we evaluated both the median and 99^th^ percentile of field strengths in the regions of pathology. The median reflects the expected performance, whereas the 99^th^ percentile reflects the risk of tissue heating. Ideally, one would aim to obtain a uniform field distribution with high median values (i.e. 200–300 V/m) and low statistical dispersion in the tumor and peri-tumor regions and, importantly, a low spatial extent of high field values beyond those regions.

Fig 8A shows the median and 99^th^ percentile of field strengths obtained in regions of pathology at different (circular) craniectomy diameters before tumor resection. Regardless of diameter, craniectomy did not affect field strength in healthy tissues to any significant extent and median field values were between 85 and 100 V/m for all configurations. However, in tumor tissue, craniectomy significantly increased the median field strength by up to 100 V/m relative to the baseline value with no craniectomy. Maximum field enhancement was obtained at a craniectomy diameter of 50 mm at which the median field strength was ~250 V/m was induced for the L/R pair. For the A/P pair, maximum enhancement was obtained at 70 mm diameter with a median field strength of ~225 V/m. At larger diameters the field intensity was nearly stable and slightly below the induced maxima. The observed effects, particularly the saturation of the field enhancement with increasing craniectomy size, were qualitatively comparable for both the L/R and A/P pairs, although the L/R pair was more efficient at all diameters of craniectomy. In the peritumoral region similar tendencies were observed, although the enhancement of field strengths with increasing craniectomy diameter was observed throughout the entire tested range for the A/P pair.

In the tumor tissue, peak field strength of ~650 V/m were obtained for the L/R pair for small craniectomies of 15–20 mm diameters (Fig 8A, bottom row). This observation reflects current funneling effects of the skull defects, which caused high current densities at the surface of the underlying intracranial tissue. These results may support the use of larger craniectomy diameters, since these distribute the current across a larger area and thus induce higher median field values across a larger segment of the tumor and peri-tumoral region, and furthermore result in lower peak field values. For the A/P pair, a similar pattern was observed, although the maximum field strength (99^th^ percentile) was considerably lower (~520 V/m) and occurred at slightly higher diameters (55 mm). In the peritumoral tissue, peak field strengths appeared to increase steadily with increasing craniectomy diameter reaching values up to 350–400 V/m depending on the active electrode array pair.

Following resection, median field strengths estimated in the peritumoral region were highly comparable to those observed before resection. Particularly, the field strength was enhanced by craniectomy up to approximately 70 mm diameter after which it stabilized at approximately 240 V/m for the L/R pair and 225 V/m for the A/P pair (Fig 8B). Peak field strengths increased up to approximately 600 V/m for the L/R pair and 450 V/m for the A/P pair both occurring at diameters of 50 mm and higher. Contrary to the situation before tumor resection, high peak field strengths were not observed at lower craniectomy diameters, likely because the high flux of charges occurring within small burr holes was distributed throughout the CSF-filled resection cavity, thereby reducing the current density and the boundary zone between CSF and the peritumoral tissue.

### Improving efficiency using multiple distributed burr holes

Since large craniectomies pose a considerable safety hazard for the patient due to impaired brain protection, clinical feasibility of the concept is therefore likely limited to craniectomies with relatively low diameters, e.g. < 40 mm. Although craniectomies in this range expectedly increase the field strength in the region of pathology their size is insufficient to obtain maximum expected benefit from the procedure in the given patient case (Subject 1). In order to assess whether stronger field enhancement could be obtained using a safer skull remodeling procedure than a large circular craniectomy we calculated the field distribution based on a model with four burr holes of 15 mm diameter distributed evenly across the superficial tumor bed or resection cavity (Fig 8C). This configuration arguably provides a better protection against blunt trauma towards the region due to the presence of protective bone bridges between the smaller burr holes. In the investigated situation with four burr holes, the total area of the skull defect was 707 mm^2^, which is entirely equivalent to single craniectomy of 30 mm diameter. The results are displayed in Fig 8A (asterisk symbols superimposed on the craniectomy graphs).

Without resection of the tumor, the use of distributed burr holes increased the median field strength in the tumor region from 211 V/m (30 mm craniectomy) to 230 V/m for the L/R pair and from 171 V/m to 204 V/m for the A/P pair (Fig 8A, upper row), without inducing higher peak field values compared to a 30 mm craniectomy (Fig 8A, bottom row). These results were quantitatively comparable to those obtained with a 50 mm craniectomy and thus higher efficacy was induced with a smaller area of skull defect. In the peritumoral region the burr hole configuration induced a field enhancement of approximately 20 V/m for both pairs relative to a 30 mm craniectomy. After tumor resection similar tendencies were observed with field enhancements in the range of 10 to 20 V/m (Fig 8B, asterisk symbols). The same range of enhancement was observed for the peak values. As observed for all other craniectomy configurations, the effects of the burr hole configuration were focal and targeted at the region underlying the skull defect. In the presented case, healthy tissues were unaffected.

Collectively, these results suggest, that it is possible to design a patterned distribution of small burr holes for the individual patient, which is both constrained to an acceptable total area and able to induce considerable enhancement of the field strength in the relevant regions. Despite the potential of this approach, a more detailed investigation of the procedure, including planning of optimal burr hole placement, is beyond the scope of the present study.

### Effect of craniectomy on deeply located tumors

**Fig 9:**
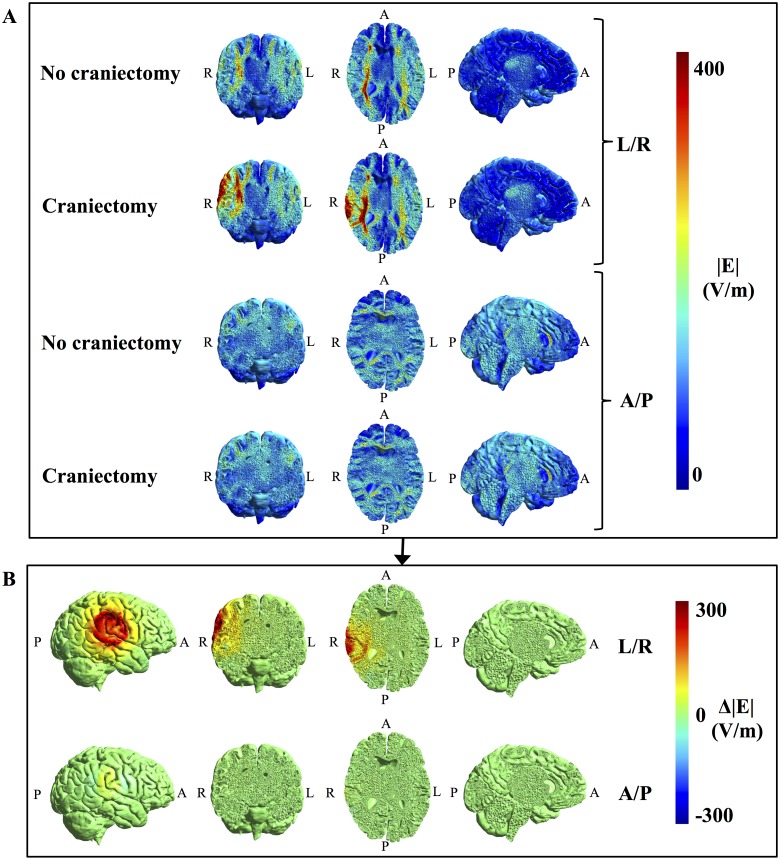
Topographical effect of craniectomy for a deeply seated tumor. **A.** Field strength distributions with and without craniectomy (coronal, axial, and sagittal sections from left to right, colorbar 0–300 V/m). **B**. Paired difference between craniectomy and no craniectomy scenarios, i.e. Δ|**E**| = |**E**|_craniectomy_—|**E**|_no craniectomy_, for both electrode pairs. Leftmost panels show a surface view of the region of pathology. Craniectomy caused no considerable changes in electrical field strength in the regions of pathology, but rather induced a significant increase in field strength in the healthy tissues immediately underlying the skull defect.

In order to evaluate the potential impact of craniectomy on tumors located deeply in the brain, analyses were performed on the model based on Subject 2 (see *Finite element head models*) who had an anaplastic astrocytoma located in the right thalamus and the surrounding regions. Deeply seated gliomas are interesting from the point of view that they are often inoperable and associated with a poor prognosis, thereby making TTFields a potentially attractive supplement to the standard regimen of radio- and chemotherapy. In this respect, it is of course also interesting to clarify whether craniectomy may potentially benefit such patients. However, as previously mentioned, craniectomy exerts its field enhancing effect by creating a corridor for enhanced current flow into the region underlying the skull defect (Fig 5) and it is questionable whether the enhancing effect will be able to reach the deeper areas. In support of this notion, results from Subject 2 indeed show that the field enhancement caused by craniectomy occurred in the healthy brain tissue underlying the craniectomy but not in the deep tumor tissue (Fig 9). Craniectomy only marginally increased the field intensity in the tumor region from 81 (IQR = 47) V/m to 91 (IQR = 19) V/m for the L/R pair while no enhancement was observed for the A/P pair with field strengths 118 (IQR = 26) V/m before craniectomy and 118 (IQR = 23) V/m after craniectomy. For the peritumoral region the results were comparable with craniectomy slightly increasing the field strength from 108 (IQR = 47) V/m to 118 (IQR = 56) V/m for the L/R pair, while no change occurred for the A/P pair with median field strength 113 (IQR = 26) V/m in both cases). As evident from Figs 4 and 9, the field enhancement induced by craniectomy was located to the region underlying the craniectomy for both Subjects 1 (tumor) and 2 (healthy brain tissue). In both cases, field enhancement occurred throughout most of the hemispheric region immediately underlying the craniectomy, albeit the most significant enhancement was observed in the cortical and subcortical structures down to a depth of approximately 4–5 cm.

On an additional note, it is also noticeable that the field induced in the deeply seated tumor (Subject 2) by standard TTFields therapy without craniectomy was considerably lower compared to the more superficial tumor (Subject 1). In the tumor region the median field intensity was 81 V/m compared to 140 V/m, respectively for the LR pair. For the A/P pair, the equivalent values were 118 V/m and 139 V/m, respectively. Similar results were obtained for the peritumoral region, although less pronounced. These results indicate, that TTFields is arguably less effective for deeply seated tumors. They also indicate, that the A/P pair contributes considerably more than the L/R pair for deep tumors, which is contrary to the superficial tumors for which the L/R and A/P pairs contributed equally. This effect likely resulted from the fact that current was effectively shunted through the lateral ventricles towards the deep tumor during activation of the A/P pair.

## Discussion

In the present work, we have evaluated the potential impact of targeted craniectomy on the estimated field distributions and efficacy of TTFields therapy. We have focused our analysis mainly on a representative case of cortical/subcortical hemispheric GBM and compared electrical field distributions calculated without craniectomy with distributions obtained following 1) removal of a realistic craniotomy bone flap, 2) introduction of a wide range of circular craniectomies of varying size all located immediately above the superficial part of the tumor, and 3) introduction of four 15 mm burr holes above the tumor. In addition, we have assessed these effects both before and after gross total resection of the tumor in order to address the impact of the approach in the most realistic clinical situations. To illustrate the potential limitations in applicability of the intervention, we have also assessed the expected efficacy on a patient with a very deeply seated tumor located in the thalamic region. All calculations were based on realistic head models constructed from structural and diffusion MRI.

### Overall efficacy of craniectomy combined with TTFields

Our results provide preliminary but strong evidence that surgical craniectomy placed in close vicinity to superficial tumors may provide a substantial and highly focal enhancement of the field strength induced by TTFields in the tumor tissue. In the present case of superficial frontoparietal GBM, standard craniectomy, i.e. removal of a realistic bone flap as could be created in connection with primary surgical tumor resection, increased in the electrical field strengths in the regions of pathology by 50–70% while leaving healthy tissues largely unaffected. In addition, standard craniectomy increased the percentage of pathological tumor tissue in expected growth arrest or regression from negligible values to more than 50% both before and following resection. When no resection was performed, craniectomy changed the expected average cancer growth pattern from considerable tumor growth to *regression* at a rate of ~10% of the normal glioblastoma growth rate. This illustrates the clinical potential of the proposed procedure and even suggests that significant disease regression might potentially be possible. Similar results were obtained with 50 mm circular craniectomy centered above the tumor center. The enhancing effect of craniectomy increased almost linearly with craniectomy diameters in the range 0–50 mm after which the effect appeared to stabilize.

Following resection of the superficial tumor the effect of standard craniectomy was qualitatively similar, although slightly less pronounced, compared to the preresection scenario. It was not possible to obtain average estimated regression of neoplastic tissue in the peritumoral region, however, craniectomy did produce a considerable (2- to 3-fold) estimated reduction of residual tumor growth rate. Again, the median field strength increased almost linearly with craniectomy diameter, in this case up to 70 mm for the L/R pair and throughout the entire range for the A/P pair.

### Mechanism of action

Craniectomy had a significant impact on the topographical distribution of the electrical field. As a general observation, the procedure induced a highly focal enhancement of the electrical field strength in the tissue lying immediately below the craniectomy (Figs 4 and 9). This effect reflected the procedure’s generic mechanism of action, namely that craniectomy created a low resistance pathway and thereby caused current to be funneled through the skull defect and into the underlying tissue (Fig 6). A high focality of field enhancement was observed for all craniectomy configurations, although less so for large diameters exceeding the superficial boundaries of the tumor region. It was observed both when no resection was performed and following gross total resection of the tumor, which indicates that the approach is likely applicable to most clinically relevant scenarios of superficial tumors.

In the case of a deeply seated tumor, craniectomy only caused a slight enhancement of the field induced by the L/R electrode pair, while no enhancement was observed for the A/P pair. This selective alteration of L/R efficiency likely resulted from the fact that the L/R electrode pair was located immediately above the skull defect and that the induced field and current vectors were perpendicular to the craniectomy plane. This caused current to flow directly into the underlying tissue. On the contrary, the A/P pair was located far from the skull defect and induced field and current vectors parallel to the hole, which therefore did not facilitate current flow into the intracranial space.

Despite the fact that craniectomy was unable to cause notable enhancement of the field in the deeply seated tumor tissue, it did indeed significantly increase the field intensity in the healthy GM and WM tissue immediately underlying the skull defect during activation of the L/R pair. This supports the notion that the intervention induces field enhancement by facilitating current flow into the underlying region. The effects of craniectomy on TTFields are therefore not related to a selective interaction with tumor tissue, but rather the location of the skull defect and electrodes relative to the tumor. In conclusion, craniectomy will expectedly be beneficial if it can be placed on the path of current flow between the active electrode and the tumor, such that currents are funneled towards the region of pathology, as illustrated in Fig 6.

### Clinical applicability

As evident from Figs 4B and 9B, the enhancing effect of craniectomy occurred down to approximately 4–5 centimeters in depth for both the superficial and deep tumor case. In addition, the anatomical range of applicability may further be extended in some situations by creating high-conductivity CSF pathways, e.g. in connection with partial or total surgical resection, such that current may be funneled from the craniectomy to deeper tumor regions. This concept is illustrated in Fig 7, which shows how gross total resection extends the topographical range of the treatment (compare to Fig 4) and increases the electrical field in deeper regions of the brain relative to the field direction. Collectively, this suggests that the intervention may potentially be beneficial for most cases of hemispheric tumors. As evident from a recent study, most GBM tumors (97%) are located in the frontal, parietal, temporal and occipital lobes of the cerebral hemispheres [25]. Only very few are located solely in deeper regions, such as the brainstem, thalami and basal ganglia. The study also showed that on average a significant proportion of the GBM tumor volume was located in the superficial most 5 cm of the cerebral hemispheres, in which the proposed intervention is expectedly beneficial. Specifically, it was shown that the median tumor volume was approximately 45 cm^3^ (≈ median radius 22 mm) and that the median tumor centroid was located approximately 50 mm from the center of the third ventricle. Both findings are highly comparable to case of Subject 1 (volume 46 cm^3^ and centroid 58 mm from middle of third ventricle). Given the generic mechanism of action, the craniectomy procedure will therefore expectedly work for a wide range of tumors located in both the frontal, parietal, temporal and occipital lobes. The concept will generally apply when 1) a significant portion of the tumor volume is located in the outermost 5 cm of the cerebral hemispheres, 2) a craniectomy can be placed in close vicinity to the region of pathology and 3) one patch in each electrode pair can be placed in close vicinity to the craniectomy. The intervention is unsuited for tumors located deeply in the thalamic regions and basal ganglia.

### Balancing safety and efficacy

For the described concept to be clinically feasible, it is necessary to carefully consider safety/efficacy profile in general terms and for each individual patient. In this regard, two major safety considerations need to be addressed: 1) What is the risk of damage to healthy tissues due to unacceptable heating, and 2) can craniectomy be planned to maintain adequate brain protection.

With regards to the first consideration, our results have confirmed the general expectation that the field enhancement caused by craniectomy was relatively focal and to a significant extent determined by the geometry and location of the craniectomy together with the position of electrodes. In the investigated cases, the most significant field enhancement was restricted to the area immediately underlying the craniectomy. In the presented case of a tumor located superficially beneath the craniectomy, no notable changes were observed in the surrounding healthy tissues. The median and peak electrical field strengths in healthy brain tissues were unaffected by craniectomy, regardless of configuration or size, as was the median SAR values in skin regions underlying the electrodes and overlying the hole in the skull (pre- and post resection of the tumor). Peak SAR values were increased by craniectomy but still within the range of median SAR values reported in previous modeling studies. Therefore the treatment would not impose additional risk of over heating or damage of healthy tissues and craniectomy would expectedly not imply safety concerns in this regard. However, single small craniectomies (15–20 mm) did appear to cause very high field strengths in the underlying tumor tissue in the pre-resection scenario. This questions the feasibility of this approach, although the affected tissue was pathological in this case and heating therefore not necessarily a critical issue. In the case of tumor resection, high peak field strengths were not observed for any craniectomy diameters, including small diameters, which indicates that craniectomy above a resected region will likely not induce heat toxicity. The mechanism behind this observation is likely that that current was dispersed more uniformly in the underlying CSF (resection cavity) which thereby reduced the current density at the tissue boundaries. Finally, it should be considered that the modeled scenarios reflect the clinical situation at a given point in time during the course of treatment. It is possible, that dynamic changes in tumor morphology and tissue properties, such as disease progression or regression, will occur during the continuous treatment with TTFields. One aspect to consider in the respect is that tumor regression, as potentially induced by TTFields or other treatment, may reduce the tumor burden in the area of maximum field intensity, such that such that generated heat could instead be deposited in the surrounding healthy tissues. For this reason, additional modeling should be performed to ensure sufficient safety if radiological changes indicate that healthy tissues could be at risk. This consideration holds for TTFields in general but particularly if the treatment is to be combined with craniectomy, as the induced field strengths in intracranial tissues are significantly enhanced by this procedure.

The second major safety concern is that craniectomy will compromise the local protection of the brain and therefore may increase the risk of traumatic brain injury. The aim is therefore to determine the smallest possible craniectomy, which provides the desired enhancement of the field strength, such that an appropriate balance between safety and efficacy is obtained. Our results suggest, that the median field strength in the region of pathology was largest for craniectomies around 50–70 mm in diameter, which is regarded as unsafe to implement without additional external skull protection. Increasing the size of the craniectomy to values larger than 50–70 mm did not appear to provide additional benefit, although this conclusion is only valid for the case investigated here and likely depends on the size and location of the tumor in question.

Despite variations between subjects, our results do imply that some craniectomies may be both safe and efficient in connection with TTFields. Even relatively small craniectomies of 30–40 mm diameter can significantly increase the field strength induced in the underlying tumor tissue and such craniectomies may be clinically safe and feasible in many cases. In addition, our results also show that even stronger field strength enhancement may be obtained be distributing the skull defect area across multiple smaller burr holes placed in the immediate vicinity of the tumor. The latter approach would likely provide better protection against blunt trauma injuries compared to single craniectomies with equivalent total area. Furthermore, the burr holes could be placed such that they could be covered directly by the ceramic transducer discs of the TTFields delivering device, thereby allowing direct current flow from the electrodes through the hole, while also providing mechanical protection over the defects.

### Limitations and future perspectives

Despite the generic mechanism of action and arguable generalizability of the results, future investigations are required to thoroughly characterize the impact of tumor location and tissue morphology/composition on the efficacy of craniectomy. Ideally, however, individualized simulations should be performed to establish the potential benefit of skull remodeling surgery for every patient before such procedures are performed. Furthermore, it would be beneficial to develop and refine the current modeling techniques for better accuracy and applicability, including estimation and validation of electrical properties for a variety of surgical products used in glioma surgery. For instance, methods that would allow for accurate modeling of the most common bone fixation implants and hemostatic products would be highly valuable in order to reproduce the field distribution in the tumor region in greater detail. Such investigations would be highly relevant for future work with TTFields modeling in general, including modeling of the treatment in its standard implementation.

It should also be noted, that the promising potential for improved disease control presented in this manuscript was based on *in vitro* evidence for a direct correlation between increased field strength and reduced tumor growth rate (therapeutic enhancement ratio) [21]. Although this relationship was established on immortalized human glioma cell lines, it may not accurately represent the interaction between TTFields and glioma cells for individual patients. It is most likely that the relationship extends to the clinical setting, however a direct correlation between the individual estimated field distribution and clinical tumor control remains to be validated. Similarly, it would be valuable to establish and quantify the correlation between field strength and TER on a case basis using cultured tumor specimens from the individual patients. Such information would potentially allow for individualized prognostication and assessment of the expected clinical benefit of TTFields for each patient.

Although many aspects of TTFields remain elusive, we do conclude that craniectomy causes a considerable enhancement of the TTFields intensity in the underlying region and that this in turn likely represents a significant enhancement of the clinical efficacy. As previously mentioned, however, it is important to consider that craniectomy is an invasive and potentially dangerous and may put the patient at additional risk. Preliminary phase 1 studies are therefore needed to characterize the clinical safety profile of the intervention and determine if the expected benefit will justify clinical implementation. Such a study is currently being planned by the Authors and the trial will expectedly initiate in late 2016. Finally, it is imperative to conduct a careful assessment of the potential risks and benefits for each individual patient before skull remodeling surgery is considered. Most importantly, such assessment should include the impact of the procedure on quality of life and subsequent treatment options. Ideally, individualized field modeling should also be performed to plan the procedure and ensure sufficient field enhancement.

## Conclusions

Our results provide preliminary but strong evidence that craniectomy located immediately above superficial intracranial tumors significantly increases the efficacy of TTFields therapy of glioblastoma by up to 100%. The concept is based on the fact that craniectomy creates a pathway of low resistance through the skull and thereby effectively guides the current towards the region of pathology. Even smaller craniectomies (30–40 mm diameter) or use of multiple burr holes (four 15 mm burr holes) may provide significant benefit while preserving acceptable brain protection and patient safety. The concept theoretically applies to a wide range of tumors and resection may extend the anatomical range of applicability. Three main criteria should be fulfilled to apply the concept: 1) a large part of the tumor should be located relatively close to the surface (4–5 cm), 2) it should be feasible to place a craniectomy immediately above the region of pathology, and 3) it should be feasible to place the TTFields electrodes in close vicinity to the craniectomy. The procedure is unsuited for deeply located tumors, such as tumors located in the brainstem, thalami and basal ganglia.

The proposed procedure may potentially provide a considerable leap forward in the treatment of one of the most serious and debilitating cancer diseases of all. Future clinical investigations are required to validate the clinical safety and efficacy of the approach.
